# Identification and Characterization of *Chlamydia abortus* Isolates from Yaks in Qinghai, China

**DOI:** 10.1155/2015/658519

**Published:** 2015-04-28

**Authors:** Zhaocai Li, Xiaoan Cao, Baoquan Fu, Yilin Chao, Jinshan Cai, Jizhang Zhou

**Affiliations:** ^1^State Key Laboratory of Veterinary Etiological Biology, Lanzhou Veterinary Research Institute, Chinese Academy of Agricultural Sciences, Lanzhou, Gansu 730046, China; ^2^Center for Animal Disease Control and Prevention in Qinghai Province, Xi'ning, Qinghai 810001, China

## Abstract

Recently, the yak population has exhibited reproductive disorders, which are considered to be associated with* Chlamydia abortus* (*C. abortus*) in Qinghai, China. In this study, a total of 9 aborted fetuses (each from a different herd) and 126 vaginal swab samples from the 9 herds were collected and analyzed.* C. abortus *DNA was detected from all of the 9 aborted fetuses and 30 of the 126 vaginal swab samples (23.81%) from yak cows in the selected herds. Four* C. abortus* strains were isolated from embryonated egg yolk sacs inoculated with foetal organ suspensions. The isolated* C. abortus* strains were further identified, which showed identical restriction profiles with the* C. abortus* reference strain using* Alu*I restriction enzyme in the RFLP test. Moreover, the isolated* C. abortus* strains and* C. abortus*-positive vaginal swab samples were genotyped by multiple loci variable number tandem repeat analysis and all belonged to the genotype 2 group. These findings suggested that* C. abortus* played a substantial role in yak abortion in Qinghai, China.

## 1. Introduction

*Chlamydia abortus* (*C. abortus*) is endemic in ruminants throughout the world.* C. abortus* can efficiently colonize the placental trophoblasts and is one of the causative agents of abortion and foetal loss in sheep, goats, and cattle in many countries [1–3]. The pathogen has also been associated with cases of abortion in pigs, horses, rabbits, guinea pigs, and mice [4] and represents a zoonotic risk to humans [5, 6]. Although this bacterium is distributed worldwide and can infect a range of hosts,* C. abortus* is considered a very homogeneous species with low genetic heterogeneity [7, 8]. A previous study has developed a multiple loci variable number tandem repeat (VNTR) analysis (MLVA) system to explore the diversity of* C. abortus*. Five selected loci with polymorphism have been shown to be suitable for genotyping of* C. abortus* strains, which results in the classification of all strains studied into six genotypes. Notably, the classification of the* C. abortus* strains into different genotypes has shown to be, to a large extent, related to their geographical origin [8].

The yak (*Bos grunniens*) is one of the most remarkable domestic animals found in the cold mountainous areas of the Himalayan region, at 3 000 meters above sea level. There are about 15 million yaks existing in the world. More than 90% of the yak population lives in the Qinghai-Tibetan Plateau, China. They provide meat, milk, wool, transportation, and home heating for the local people. Thus, the yak is the basis for much of the agricultural economy of the vast rural regions where few other domestic animals will survive. In general, female yaks can only be pregnant once every 2 years or twice in 3 years, and each yak cow produces a single offspring. In addition to the low reproductive rate, yaks are suspected to be infected with a large variety of pathogens including* Chlamydia* [9, 10], which significantly affect the production of yak resulting in great economic losses. Several studies have shown the seroprevalence of* Chlamydia* infection in yak herds with abortions in different regions in China [11, 12]. Therefore, the aim of the present study is to identify and characterize the* C. abortus* strains prevalent in yaks in Qinghai, China.

## 2. Materials and Methods

### 2.1. Samples

During the birth season of 2012, yak flocks in several regions of Qinghai province, China, have demonstrated the problem of abortion. A serological analysis suggested that* Chlamydia* infection was incriminated. For further diagnosis, a total of 9 samples from aborted fetuses in Guinan County (six herds) and Haiyan County (three herds), Qinghai province, each of which was from different herds, were sent to Center for Animal Disease Control and Prevention in Qinghai province and Lanzhou Veterinary Institute for further diagnosis. Another 126 vaginal swabs were taken from yak cows in these 9 herds, some of which were taken from yak cows that suffered from abortion, and the others were taken from pregnant yak cows that had not delivered or aborted yet.

### 2.2. PCR Analysis

DNA was isolated from fetal tissues and vaginal swab samples using the QIAamp DNA Kit (Qiagen) according to the kit's instructions. The DNA samples were then subjected to PCR detection for* Chlamydia*. The genus-specific primers were targeted to the* pmp* gene of* Chlamydia* (FP: 5′-ATGAAACATCCAGTCTACTGG-3′; RF: 5′-TTGTGTAGTAATATTATCAAA-3′) and PCR reaction was performed as described previously [13].

### 2.3. Isolation of* Chlamydia* Strain

For isolation, a 10% (v/v) suspension of foetal organ samples (liver, spleen, and lung) was prepared in SPG buffer (0.25 M sucrose, 10 mM sodium phosphate, and 5 mM L-glutamic acid) containing 1 mg/mL of streptomycin and 1 mg/mL of kanamycin. 0.4 mL of the suspension was inoculated into the yolk sac of 7-day-old specific pathogen free embryonated chicken eggs. The eggs were incubated at 37°C and observed daily and were harvested while they died during 4–10 days after inoculation for further passage. Blind passages were made when no embryonic death had been observed. The presence of chlamydial progeny in yolk sacs was detected by* C. abortus*-specific PCR. To identify the chlamydial inclusion body, positive cultures were further inoculated in McCoy cells monolayer, followed by Giemsa staining.

### 2.4. PCR-RFLP Analysis

For identification of the isolated* Chlamydia* strains, PCR-RFLP analysis of the helicase genes clone 8 and* omp2* gene was performed as described previously [14, 15]. The reference strains of* C. abortus* SX5 (isolated from cattle),* C. psittaci* CpL (isolated from chicken), and* C. pecorum* E58 were kept in our laboratory [16, 17]. Amplified DNA fragments of helicase genes clone 8 and* omp2* were digested with 3 U of* Alu*I in 30 *μ*L final volume overnight at 37°C, respectively. The resulting fragments were subjected to electrophoresis in 2% agarose gel at 60 V for 2 h and examined under UV illumination.

### 2.5. MLVA Typing of the Isolates and Clinical Samples

A set of VNTR loci, namely, ChlAb_300, ChlAb_457, ChlAb_581, ChlAb_620, and ChlAb_914, were used as the genotyping markers. PCR was performed to amplify the tandem repeat sequences from the* C. abortus* isolates and the* Chlamydia* DNA positive vaginal swab samples, respectively. The primers and PCR procedure were described previously [8]. The obtained amplicons were then sequenced at Shanghai Sangon Biotech (Shanghai, China). The numbers of repeats in the set of VNTR loci for each* Chlamydia abortus* isolate or sample were recorded. Therefore, an allelic profile for each* C. abortus* isolate or vaginal swab sample was obtained as an ordered string of allele numbers corresponding to the number of repeat units at each MLVA locus. To determine the MLVA genotype, the assessed allelic profile was compared with the reference genotyping data [8].

## 3. Results

### 3.1. *Chlamydia* Related Abortion

**Figure 1:**
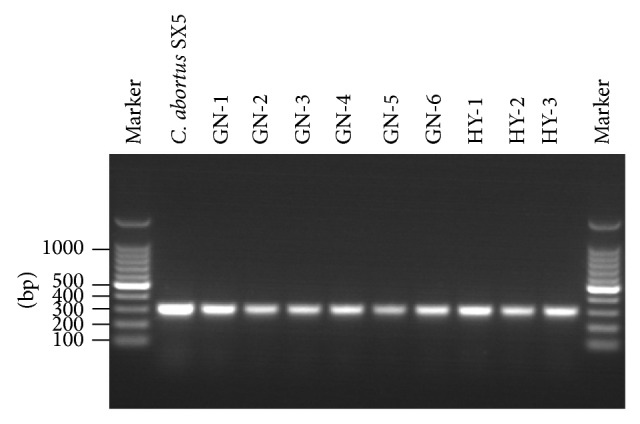
Detection of* Chlamydia* DNA from the 9 aborted yak fetuses from different herds. Marker: 100 bp DNA ladder.

During April and May of 2012, yak flocks have demonstrated the problem of abortion in several regions of Qinghai province, China. The abortion occurred at the end of gestation (1~2 months before delivery) without any premonitory signs. A serological analysis of several abortion diseases performed on the female's serum sample suggested that* Chlamydia* was incriminated (data not shown). In the present study, we investigated the 9 aborted fetuses and 126 vaginal swab samples from corresponding herds by* Chlamydia* genus-specific PCR analysis. The* Chlamydia* DNA was found in all of the aborted fetuses (Figure 1).* Chlamydia* DNA was also present in 30 of the 126 vaginal swab samples (23.81%) from yak cows in herds with abortion (Table 1). The results suggested that the yak abortion might be associated with* Chlamydia* infection.

### 3.2. Isolation and Identification of Yak* C. abortus* Isolates

**Figure 2:**
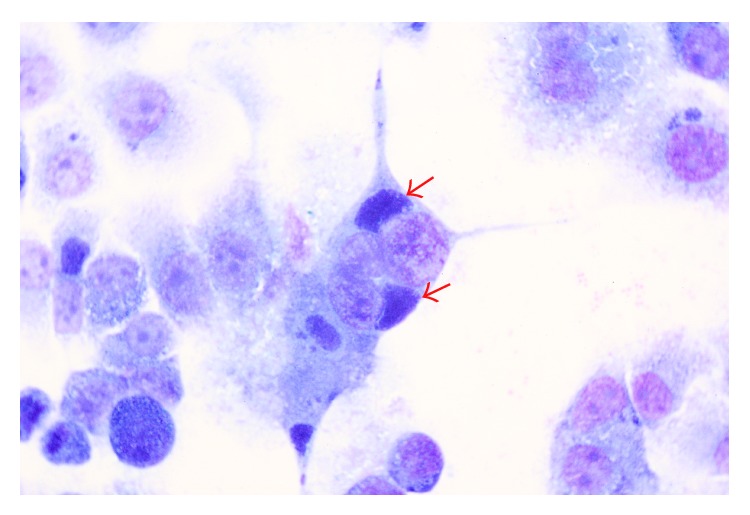
Perinuclear chlamydial inclusions (arrows) in McCoy cells when infected with the isolated strain GN-6. ×400, Giemsa.

After several sets of passages, four yak* Chlamydia* strains were isolated from the aborted yak foetal organ samples using embryonated chicken egg yolk sac culture, as evidenced by the presence of* C. abortus* DNA when examined by PCR assay. To identify the chlamydial inclusion body, the positive cultures were inoculated in McCoy cells monolayer, followed by Giemsa staining.  Figure 2 showed the chlamydial inclusions in McCoy cells after 48 h of inoculation. The isolated strains were named as GN-1, GN-4, GN-6, and HY-1 according to their origin region and herd identifier. No* Chlamydia* strain could be isolated from the other aborted yak fetuses since no* C. abortus* DNA could be detected from the yolk sacs of embryonated eggs after a series of 5 passages.

**Figure 3:**
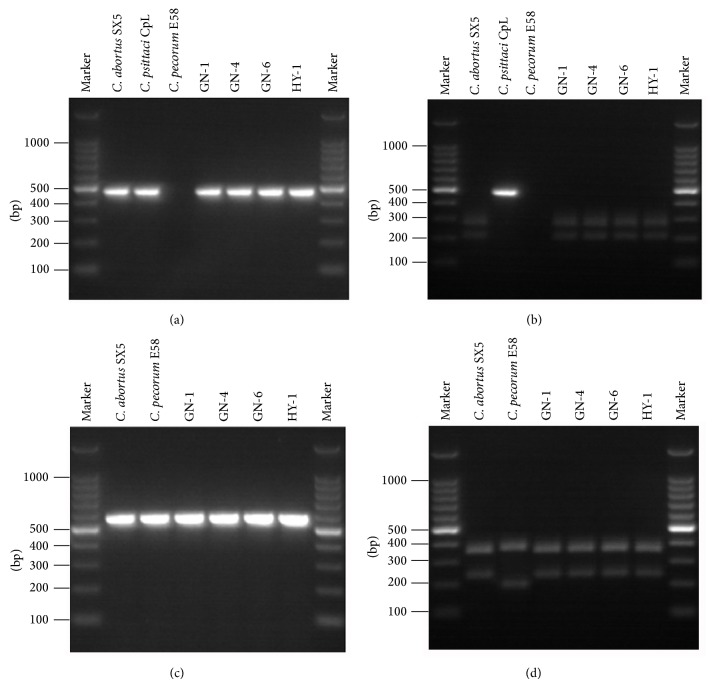
Identification of* C. abortus* isolates by PCR-RFLP analysis. (a) Helicase gene clone 8 PCR products. (b) RFLP analysis by* Alu*I restriction enzyme of clone 8 PCR products. (c)* omp2* PCR products. (d) RFLP analysis by* Alu*I of* omp2* PCR products. Marker: 100 bp DNA ladder.

For further species identification of the four isolates, amplicons of helicase genes clone 8 and* omp2* gene were subjected to PCR-RFLP analysis. As shown in Figure 3, PCR using helicase genes clone 8 primers produced a band of 479 bp from the four isolates and reference strain of* C. abortus* SX5. A positive band was also observed from the* C. psittaci* CpL; however, no band was observed from the* C. pecorum* E58 (Figure 3(a)). When the amplified DNA fragments of helicase genes clone 8 were digested with the* Alu*I enzyme, all isolates exhibited a profile with two bands of 271 bp and 208 bp, the same as that of the* C. abortus* SX5 reference strain. However, the intact amplified fragment was observed without any cleavage from* C. psittaci* CpL strain after* Alu*I enzyme digestion, which differentiated it from the four yak isolates (Figure 3(b)). PCR using* omp2* primers produced a band of 587 bp from the four isolates and reference strains of* C. abortus* SX5 and* C. pecorum* E58 (Figure 3(c)). Following digestion with the* Alu*I enzyme, all isolates exhibited the same profile with two bands of 352 bp and 235 bp as the* C. abortus* SX5 strain, while* C. pecorum* E58 exhibited a different profile with two bands of 397 bp and 193 bp (Figure 3(d)). Therefore, the yak isolates were identified as* C. abortus* strains.

### 3.3. MLVA Genotyping of* C. abortus* Isolates and Clinical Samples

In this study, we determined the genotype of yak* C. abortus* isolates and the 34 clinical samples by using the MLVA typing method. The five polymorphic loci, namely, ChlAb_457, ChlAb_581, ChlAb_620, ChlAb_914, and ChlAb_300, were amplified and sequenced, and the repeated units in each locus were recorded. The results showed that the same allelic profile (1-1-2-1-3) was shared by all the isolates and clinical samples, which matched the MLVA genotype 2 of* C. abortus* (see Figure S1 in Supplementary Material available online at http://dx.doi.org/10.1155/2015/658519 and Table 1) [8]. Therefore, we classified the yak isolates into MLVA genotype 2. The results may also suggest that the MLVA genotype 2 of* C. abortus* is the predominant strains prevalent in yaks in Qinghai, China.

## 4. Discussion

The yak populations seem to be susceptible to most pathogenic agents that affect cattle. Serological studies showed that* Brucella*,* Toxoplasma gondii*, and bovine viral diarrhea virus infection in yaks commonly occurred in Qinghai province, China [18–20]. In recent years,* Chlamydia* infection has attracted attention. Several Chinese literatures showed a seroprevalence rate (9.37%~57.14%) in yak populations in different areas of Qinghai-Tibet Plateau [21, 22]. As early as 1988, Shuai et al. reported that abortions in yaks in a part of Qinghai province were diagnosed as being caused by* Chlamydia abortus* infection [23]. Ma surveyed the prevalence of* Brucella* and* Chlamydia* in Qinghai province in 2002, and the results showed the incidence of abortion in yak due to* Chlamydia* may be of significance [9]. However, there has been no direct detection or isolation of Chlamydiae from yaks. In this study, we succeeded to isolate and characterize chlamydial strains responsible for yak abortion. The 9 aborted fetus samples were analyzed by species-specific PCR for* C. abortus*. DNA of* C. abortus* was detected from all of the samples, suggesting that* C. abortus* infection was responsible for the yak abortion cases. The inoculation of infected foetal organ extract into chicken embryos led to the isolation of four* C. abortus* strains. Some reports also showed that, in addition to* C. abortus*,* C. psittaci* and* C. pecorum* were also associated with cases of abortion in sheep and goat [24, 25]. Due to the high degree of phenotypic similarity and for further identification of the isolated strains, it is necessary to differentiate the species from each other. It has been suggested that* C. abortus* can be differentiated from* C. psittaci* and* C. pecorum* by RFLP analysis of PCR products obtained with primers specific to clone 8 of the* C. abortus* helicase gene and* omp2* gene and that these primers allow species-specific identification [14, 26]. The present study showed that PCR-RFLP with clone 8 and* omp2* primers is adequate for the species identification of* C. abortus* and the result further confirmed that the yak abortion cases were associated with* C. abortus* infection.

*C. abortus* is an obligate intracellular bacterium which can colonize very different types of placenta (ruminant, porcine, murine, and human), causing abortion during the last third of the gestation period. This disease is especially important in yaks, one of the natural hosts in Qinghai-Tibet Plateau, because of the economic losses it causes. However, chlamydial diseases in yaks are not well understood, partially because they have received relatively little attention. In addition, the bacteria also present a potential zoonotic risk to pregnant women, since several cases of human abortion have been reported as being associated with exposure to aborted sheep or goat caused by* C. abortus* [5, 6]. Therefore, there is a clear need for improved control strategies for this issue including more effective diagnosis and control measures. For diagnosis, isolation of the pathogens from aborted samples (fetuses and placentae) represents the gold standard for definitive diagnosis. However, isolation of bacteria requires obtaining samples in optimal conditions (they must be fresh, with little or no contamination, and free of toxic factors) that contain a threshold number of live and viable microorganisms. This process is labor intensive and time consuming and therefore not suitable for routine diagnostics or large-scale epidemiological investigations. Indirect hemagglutination (IHA) test based on the* C. abortus* elementary bodies is the most common and available reagent for routine diagnosis of the* C. abortus* antibodies in China. However, cross-reactions with* C. psittaci* and* C. felis* can be observed [27, 28]. Detection of* Chlamydia* specific DNA by PCR is more sensitive and specific than serological tests and therefore recommended. Conventional and real-time PCR have been widely used to identify* C. abortus* in clinical samples. Further study is now necessary to get a better understanding of the epidemiology of the bacterium in the yak populations by integration of these techniques in routine diagnostics.

For prevention, vaccination may be the most effective way of prevention of* C. abortus* related abortion. A live, avirulent* C. abortus* 1B vaccine has been developed and found to suppress abortions and prevent the persistence of subclinical infection in sheep and goat [28]. In China, inactivated vaccines against* C. abortus* for sheep and cattle were developed and used in several regions such as Gansu, Xinjiang, Yunnan, Inner Mongolia, Ningxia, and Shaanxi [16, 29]. However, up to now, there is no effective vaccine available for yak chlamydiosis. It is worth noting that the same vaccination measure may present different efficacies against different* Chlamydia abortus* strains due to genetic heterogeneity between the vaccine and target strains. For instance,* C. abortus* strains such as LLG and POS isolated in Greece seem to display genetic heterogeneity and exhibit phenotypic differences in antibody cross-reactivity [30]. The live 1B vaccine was more effective against the AB16 strain than against the LLG and POS strains [31]. In addition, the strains 1B and AB16 belonged to MLVA genotype 2, while LLG and POS strains belonged to MLVA genotype 6 [8]. Therefore, the genetic characters of the local epidemic strains should be clearly understood when developing a new vaccine or vaccination. In the present study, we analyzed the genotype of the* C*.* abortus* isolates and field samples by MLVA as described previously [8]. The result showed that both the isolates and the field epidemic strains all belonged to the same MLVA genotype, genotype 2. This may imply that field epidemic strains share a similar genetic background and that vaccination measures targeting the most prevalent serotype might be effective for most of the yak herds in Qinghai, China.

## 5. Conclusions

The results from PCR and bacteria isolation showed that* C. abortus* is responsible for yak abortion in some areas of Qinghai province, China. It is necessary to carry out further research on prevalence and distribution of* C. abortus* to better understand its impact in yak health and production in Qinghai-Tibet Plateau. Pertinent measures to control and prevent* C. abortus* transmission among animals also need to be implemented.

## Supplementary Material

The MLVA typing method was used to determine the genotype of yak *C. abortus* isolates and the 34 *Chlamydia* DNA positive clinical samples. The five polymorphic loci, namely ChlAb_457, ChlAb_581, ChlAb_620, ChlAb_914 and ChlAb_300, were used as the genotyping markers. They were amplified and sequenced, and the repeated units in each locus were recorded. An allelic profile for each *C. abortus* isolate or vaginal swab sample was obtained as an ordered string of allele numbers corresponding to the number of repeat units at each MLVA locus. The results showed that a same allelic profile (1-1-2-1-3) was shared by all the isolates and clinical samples, which matched with the MLVA genotype 2 of *C. abortus*. Therefore, we classified the yak isolates and prevalent strains into MLVA genotype 2.

## Figures and Tables

**Table 1:** Detection of *C. abortus* by PCR from vaginal swab samples.

Herds	Herd size	Number of vaginal swabs	Positive PCR: number (and %)	MLVA genotype of the positive samples
GN-1	340	21	4 (19.05)	2
GN-2	203	12	2 (16.67)	2
GN-3	280	18	3 (16.67)	2
GN-4	267	15	5 (33.33)	2
GN-5	271	10	4 (40.00)	2
GN-6	189	7	4 (57.14)	2
HY-1	334	19	6 (31.58)	2
HY-2	265	11	1 (9.09)	2
HY-3	310	13	1 (7.69)	2

Total		126	30 (23.81)	
