# Supplementary material for: Identification and Characterization of Chlamydia abortus Isolates from Yaks in Qinghai, China
Source: Biomed Res Int. 2015 Apr 28;2015:658519. doi: 10.1155/2015/658519 (PMC4427853; doi:10.1155/2015/658519)
Supplement: Supplementary file 1 — The MLVA typing method was used to determine the genotype of yak C. abortus isolates and the 34 Chlamydia DNA positive clinical samples. The five polymorphic loci, namely ChlAb_457, ChlAb_581, ChlAb_620, ChlAb_914 and ChlAb_300, were used as the genotyping markers. They were amplified and sequenced, and the repeated units in each locus were recorded. An allelic profile for each C. abortus isolate or vaginal swab sample was obtained as an ordered string of allele numbers corresponding to the number of repeat units at each MLVA locus. The results showed that a same allelic profile (1-1-2-1-3) was shared by all the isolates and clinical samples, which matched with the MLVA genotype 2 of C. abortus. Therefore, we classified the yak isolates and prevalent strains into MLVA genotype 2. [file 658519.f1.doc]

**Supplementary figure**


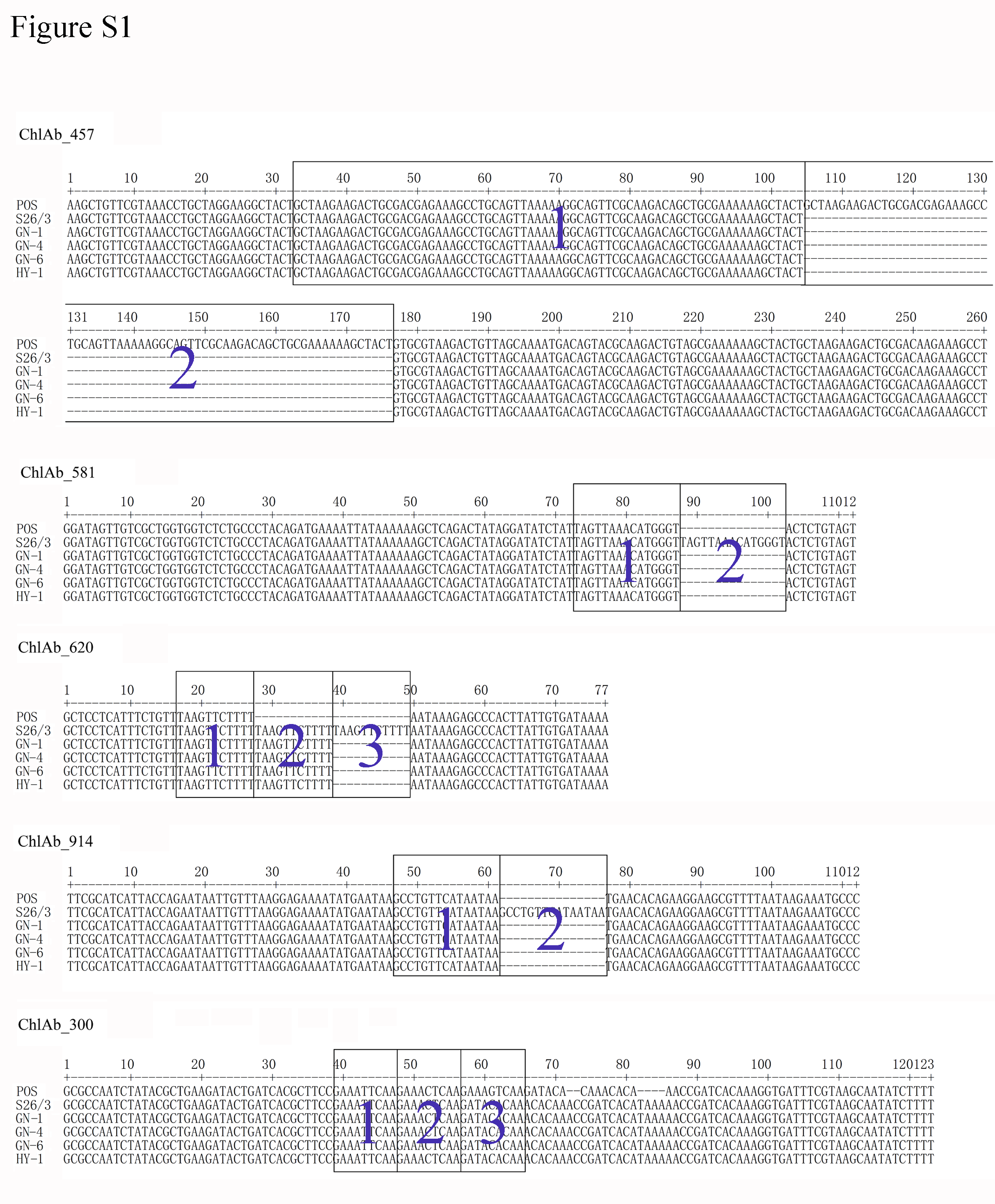


Figure S1. VNTR localizations and the number of repeat units. MLVA genotyping markers ChlaAb_457, ChlaAb_581, ChlaAb_620, ChlaAb_914 and ChlaAb_300 were amplified from the yak *C. abortus* isolates and were sequenced. The obtained sequences were aligned with that of the reference strains POS and S26/3 [8]. The repeat copies are boxed and marked.
